# Deciphering the Gene Regulatory Landscape Encoded in DNA Biophysical Features

**DOI:** 10.1016/j.isci.2019.10.055

**Published:** 2019-10-31

**Authors:** Abhijeet Pataskar, Willem Vanderlinden, Johannes Emmerig, Aditi Singh, Jan Lipfert, Vijay K. Tiwari

**Affiliations:** 1 Netherlands Cancer Institute, Amsterdam, the Netherlands; 2 Department of Physics and Center for NanoScience, LMU Munich, 80799 Munich, Germany; 3 Wellcome-Wolfson Institute for Experimental Medicine, School of Medicine, Dentistry & Biomedical Science, Queens University Belfast, Belfast BT9 7BL, UK; 4 Former Address: Institute of Molecular Biology, 55128 Mainz, Germany

**Keywords:** Genetics, Molecular Genetics, Biophysics

## Abstract

Gene regulation in higher organisms involves a sophisticated interplay between genetic and epigenetic mechanisms. Despite advances, the logic in selective usage of certain genomic regions as regulatory elements remains unclear. Here we show that the inherent biophysical properties of the DNA encode epigenetic state and the underlying regulatory potential. We find that the propeller twist (ProT) level is indicative of genomic location of the regulatory elements, their strength, the affinity landscape of transcription factors, and distribution in the nuclear 3D space. We experimentally show that ProT levels confer increased DNA flexibility and surface accessibility, and thus potentially primes usage of high ProT regions as regulatory elements. ProT levels also correlate with occurrence and phenotypic consequences of mutations. Interestingly, cell-fate switches involve a transient usage of low ProT regulatory elements. Altogether, our work provides unprecedented insights into the gene regulatory landscape encoded in the DNA biophysical features.

## Highlights

• DNA shape features encode genomic surface accessibility and flexibility
• High ProT is a deterministic feature of enhancers
• ProT levels correlate with nuclear organization of epigenetic states
• Cell-fate switches involve a transient usage of low ProT regulatory elements

## Graphical Abstract

**Figure:**
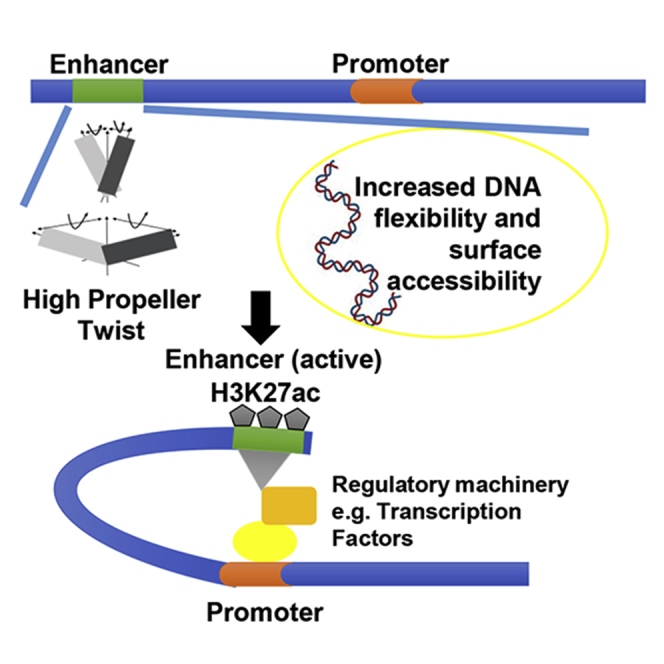

## Introduction

The genome consists of multiple gene regulatory units comprising of proximal and distal regulatory elements. Recent studies have shown that the function and utilization of these elements during cellular differentiation and in response to intracellular and extracellular cues relies on a dynamic control by epigenetic machinery in concert with transcription factors (TFs) (Li et al., 2007, Long et al., 2016, Margueron and Reinberg, 2010, Moris et al., 2016). Enhancers are known to be critical for setting up transcriptome underlying cell-type identity and function from far away distances on their target promoters (Heinz et al., 2015, Long et al., 2016, Rickels and Shilatifard, 2018, Sakabe et al., 2012, Schoenfelder and Fraser, 2019, Shlyueva et al., 2014, Spitz and Furlong, 2012). Importantly, mutations in these gene regulatory elements are known to disrupt their function affecting gene expression and ultimately cell identity and hence underlie several diseases (Li et al., 2019, Rickels and Shilatifard, 2018, Sakabe et al., 2012, Weinhold et al., 2014). Typically, a range of methods are employed to identify and validate such distal regulatory elements including quantifying certain histone modifications and DNase hypersensitivity assays (Ong and Corces, 2011, Pradeepa et al., 2016, Shlyueva et al., 2014, Zentner and Henikoff, 2013). These methods have their own limitations and a number of alternate assays and histone modifications have recently been used to discover enhancers (Arnold et al., 2013, Pradeepa et al., 2016, Vanhille et al., 2015). Thus, our current approach to reveal regulatory elements in entirety is highly limited and vouches to search for conserved features that can explain enhancer evolution and function.

Interestingly, sequences from regulatory loci are able to recapitulate endogenous TF binding pattern, chromatin state, and cell-type-specific activity when placed at an exogenous genomic site or tested in isolation (Lienert et al., 2011, Yanez-Cuna et al., 2013). In addition, computational analysis has further shown that the occurrence of certain sequences at genomic loci is predictive of their regulatory potential (Colbran et al., 2017, Yanez-Cuna et al., 2014, Yang et al., 2017a). These lines of evidence strongly suggest the existence of inherent gene regulatory potential of these genomic loci at the sequence level. Despite these advances, we lack understanding of the evolutionary constrains in the selection of certain genomic DNA elements for their gene regulatory function (Pennacchio et al., 2013). It is thus important to decode the power of sequence features in determining the gene regulatory potential and differential usage in cell-type specification. In addition, it is important to catalog novel regulatory elements, an effort that is limited by insufficient knowledge of existing features of these elements.

Several laboratories have attempted to employ computational approaches to predict enhancers based on sequence information (Kleftogiannis et al., 2016, Lee et al., 2011, Rusk, 2014). Although these methods were able to predict enhancers to a certain degree, they were unable to decipher the underlying code that drives enhancer selection and strength (Pennacchio et al., 2013). A previous study suggested that the local DNA topography differs at functional noncoding regions of the genome including enhancers (Parker et al., 2009). Interestingly, DNA shape features such as propeller twist (ProT), major grove width, and helical twisting determine different local geometries, which in turn contribute to the control of transcription factor binding and gene regulation (Greenbaum et al., 2007, Ma et al., 2017, Mathelier et al., 2016, Zhou et al., 2013). Overall, the existing evidences suggest a genetic feature code beyond simple sequence that may dictate selection of enhancers and their strength of function. Here we show that DNA shape features are highly informative of the gene regulatory potential of genomic loci. We discover that the ProT levels can reveal the location of enhancers, their strength, the affinity landscape of transcription factors, and distribution in the nuclear 3D space with high accuracy. Using experimental assays including single-molecule AFM imaging measurements, we show that indeed high ProT levels cause increased DNA flexibility and surface accessibility and may potentially explain their usage as regulatory elements. Furthermore, ProT levels also determine the effectivity landscape of the genome to tolerate mutations. Altogether, this work reveals the gene regulatory landscape encoded in the basic genetic sequence features and provides a significant advance in unfolding the mysteries of genetic code.

## Results

### Genomic Surface Accessibility and Flexibility Are Encoded in DNA Shape Features

**Figure 1:**
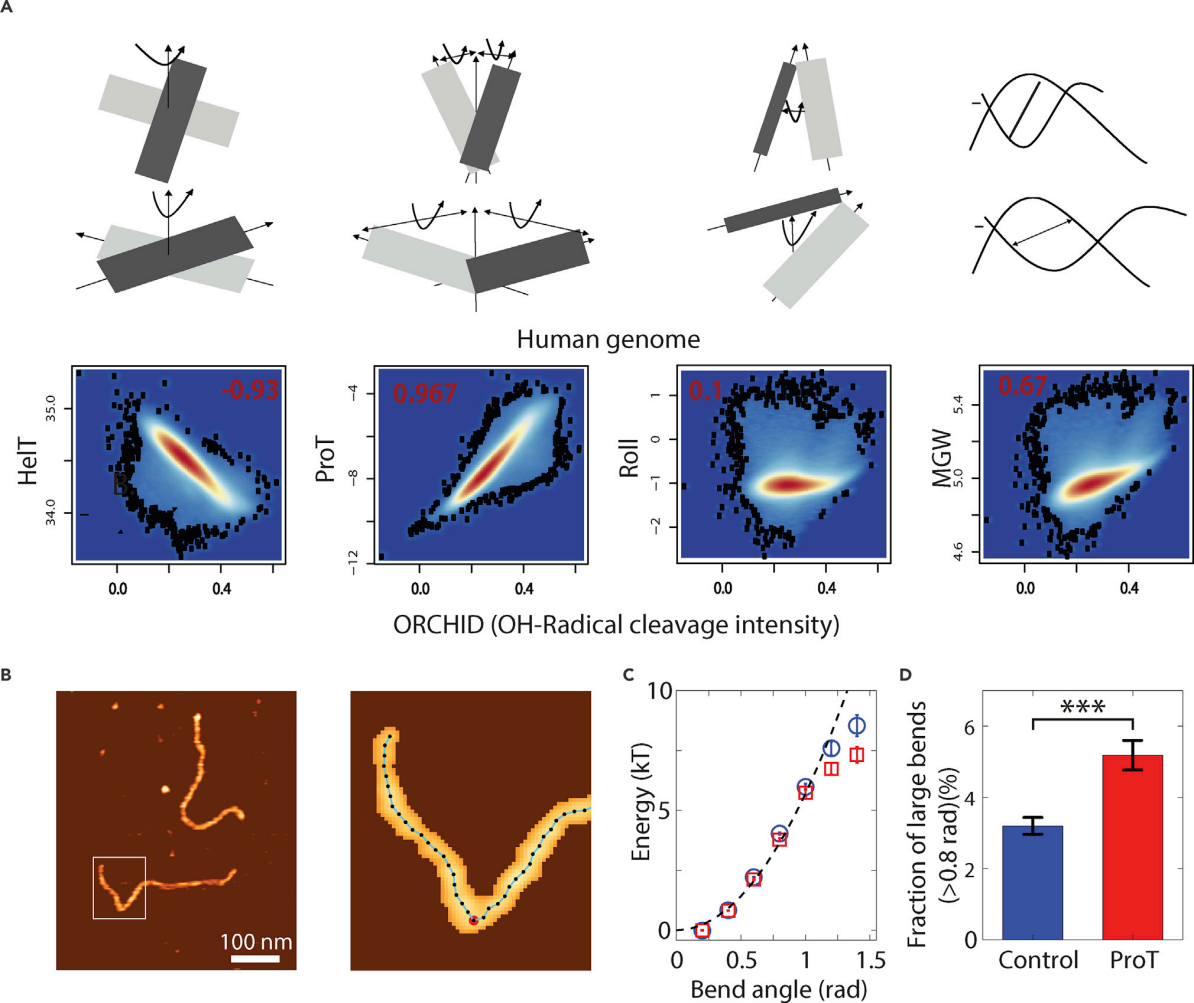
DNA Surface Accessibility and Flexibility Are Encoded in Its Biophysical Features (A) Human genome-wide correlations of predicted values of DNAshape features; helix turn (HelT), propeller twist (ProT), roll (Roll), and major groove width (MGW) with surface accessibility of the DNA as measured by OH-radical cleavage intensity predictions. (B) Typical AFM topographic image depicting two linear DNA molecules (left). Enlarged view of the boxed area (left) superimposed with the traced contour (right). The red point highlights the location of a large bend (>0.8 rad). (C) Energy landscape for bend angles reconstructed from the bend angle distribution for pooled control (*N*_*molecules*_ = 801) and pooled ProT (*N*_*molecules*_ = 425) sequences. In total, we traced 87,168 bend angles from 1,226 imaged DNA molecules. The broken line depicts the energy landscape expected for a worm-like chain with persistence length P = 55 nm. (D) Fraction of large bends (>0.8 rad) for pooled control (*N*_*large bends*_ = 182; *N*_*bends, total*_ = 56,874) and high ProT (*N*_*large bends*_ = 157; *N*_*bends, total*_ = 30,294) sequences. The fraction of large bends is significantly higher for the ProT versus control sequences (p = 7.5 × 10^-6^). The error bar is the standard deviation from counting statistics, i.e. the square root of the counts divided by the number of total counts. See also Figure S1.

The ability for genomic regions to function as gene regulatory elements is thought to be significantly influenced by their inherent accessibility for DNA-binding proteins such as TFs (Bell et al., 2011). To probe accessibility, we began by investigating whether the surface accessibility of DNA is influenced by its biophysical features. We used hydroxyl radical cleavage maps as a proxy for solvent accessible surface area of the DNA (Greenbaum et al., 2007) and correlated this with various DNA shape features such as ProT, major groove width (MGW), helix turn (HelT), and roll predicted by an established tool—DNAshape (Zhou et al., 2013). We found that ProT, defined as the angle of twisting of two neighboring nucleotides from the axis of their geometrical center, highly correlates with the DNA surface accessibility (Pearson correlation coefficient = 0.967, pval <0.001) (Figure 1A). The other features do not show as strong a correlation with hydroxyl radical cleavage maps and with each other (Figures S1A–S1C). This analysis established ProT as a proxy to measure inherent surface accessibility of DNA.

Next, to directly test how increased levels of ProT affect the mechanical properties of DNA segments, we decided to carry out high-resolution AFM imaging experiments of ∼1 kbp long DNA sequences with different ProT levels (Data S1). Toward this, we first used PCRs to generate different linear sequences predicted to be either genomic “random” or “high” in terms of ProT levels. Subsequently, atomic force microscopy (AFM) images of the DNA molecules were measured and analyzed by tracing the DNA paths (Figure 1B). An analysis of the mean-squared separation of pairs of points located at different distances along the contour length confirmed that the DNA molecules were equilibrated at the surface (Figure S1D) and allowed determination of the bending persistence length. We found bending persistence length values P ≈ 56 nm, in good agreement with previous measurements under similar conditions (Mazur and Maaloum, 2014, Rivetti et al., 1996, Wiggins et al., 2006). The data did not reveal significant differences in the persistence lengths from control (P = 56.1 ± 0.2 nm) and high ProT sequences (P = 56.7 ± 0.6 nm), suggesting that the bending stiffness at longer length scales is similar for different levels of ProT.

Therefore, to probe the local flexibility of the DNA sequences, we analyzed the distribution of bend angles between points separated by 5 nm along the contour. Taking the negative logarithm of the histogram of bend angles directly gives the effective bending energy (Figure 1C). For both random and high ProT sequences, the data for angles up to θ ∼ 1 rad are well described by a simple elastic model, the so-called worm-like chain, whereas for bending angles θ > 1 rad clear deviations from the elastic model are apparent, as have been observed previously (Wiggins et al., 2006). Interestingly, the high ProT sequences exhibited larger deviations from the elastic model and a significantly higher fraction of medium (θ > 0.8 rad; p = 7.5·10−6) and large bends (θ > 1.1 rad; p = 0.0013) compared with the control sequences (Figure 1D). In contrast, different control sequences and different high ProT sequences gave the same fractions of medium and large bends, respectively, within experimental error. Taken together, the AFM imaging analysis suggested that on short length scales (∼5 nm), high ProT sequences exhibit enhanced bendability compared to random sequences.

### Propeller Twist Levels Correlate with 3D Nuclear Positioning of Distinct Chromatin States

**Figure 2:**
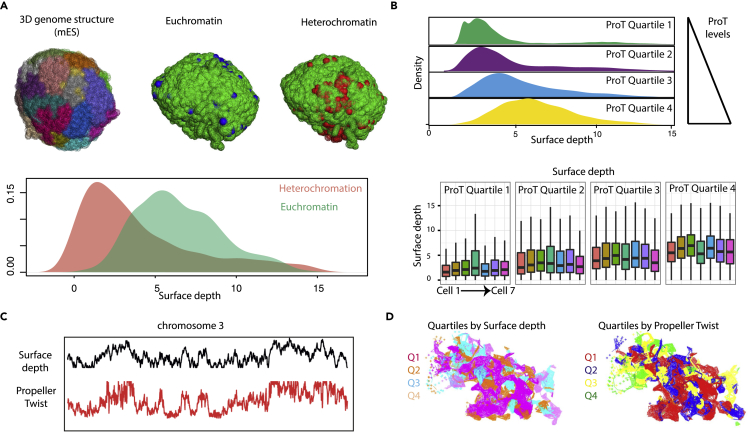
Propeller Twist Predicts 3D Nuclear Positioning Hallmark of Distinct Chromatin States (A) Upper panel: (Left) chromosomes overlaid in different colors in reconstructed single cell genome structure with the resolution of 1MB from single cell HiC studies in mouse ES cells (Stevens et al., 2017). (Mid and Right) Genomic subunits each of 1MB highlighted in blue and red depending of enrichment of euchromatin (blue) and heterochromatin (red) features. Lower panel: density plot depicting surface depth in the reconstructed genome structures of euchromatin feature and heterochromatin features. (B) Density plot depicting enrichment of surface depth from reconstructed genome structure of mES cell into genome subunits characterized into four quartiles in increasing amount of median Propeller Twist (ProT) values arranged from top to bottom. Lower panel: boxplots depicting surface depth values for every quartile of ProT values in all seven different studies single cell genome structures. (C) Line plot depicting profiles for ProT (red) and surface depth (Cell 1, black) aimed toward displaying linear correlation of these two features across chromosome 3. (D) Reconstructed 3D structure of chromosome 3 color-overlaid with quartiles of Surface depth (left) and ProT levels (right). See also Figure S2.

Eukaryotic genomes are compartmentalized into distinct domains marked by active (eu-) and inactive (hetero-) chromatin. Inspired by the observation that ProT highly correlates with the inherent surface accessibility and bendability of DNA, we hypothesized that these regions could potentially mark open active chromatin regions that are also known to be more fluid in nature. A previous study employed single cell Hi-C assays to reconstruct the 3D genome of mouse embryonic stem (mES) cells at a high resolution (Stevens et al., 2017). We processed these data and overlaid with histone modifications indicative of euchromatin and heterochromatin, H3K27ac and H3K9me3, respectively. Interestingly, euchromatin was found to have a higher surface depth (as defined in Stevens et al., 2017) as compared with heterochromatin (Figures 2A and S2A). These findings are also in line with the local enrichment of heterochromatic lamina-associated domains (LADs) at the nuclear periphery (van Steensel and Belmont, 2017).

We next analyzed the radial distribution of sequences with different ProT levels within the Hi-C data derived from 3D nuclear positioning. Interestingly, ProT levels were found to correlate well with the nuclear distribution, where “high ProT” sequences occupy an internal position, whereas “low ProT” sequences are localized at the periphery (Figure 2B). Importantly, while the surface depths of genomic loci at the single cell level across various ES cells is variable, the overall radial positioning of differential ProT regions in the genome is highly consistent (Figures S2B, S2C, and 2B). Simultaneous visualization of chromosome-wide surface depth and ProT profiles also showed a clear correlation between these two features (Figures 2C, 2D, and S2D). Collectively, these results suggested a potential contribution of ProT in influencing the nuclear positioning and its association with distinct epigenetic states.

### Propeller Twist Encodes the Regulatory Potential of Genetic Elements

**Figure 3:**
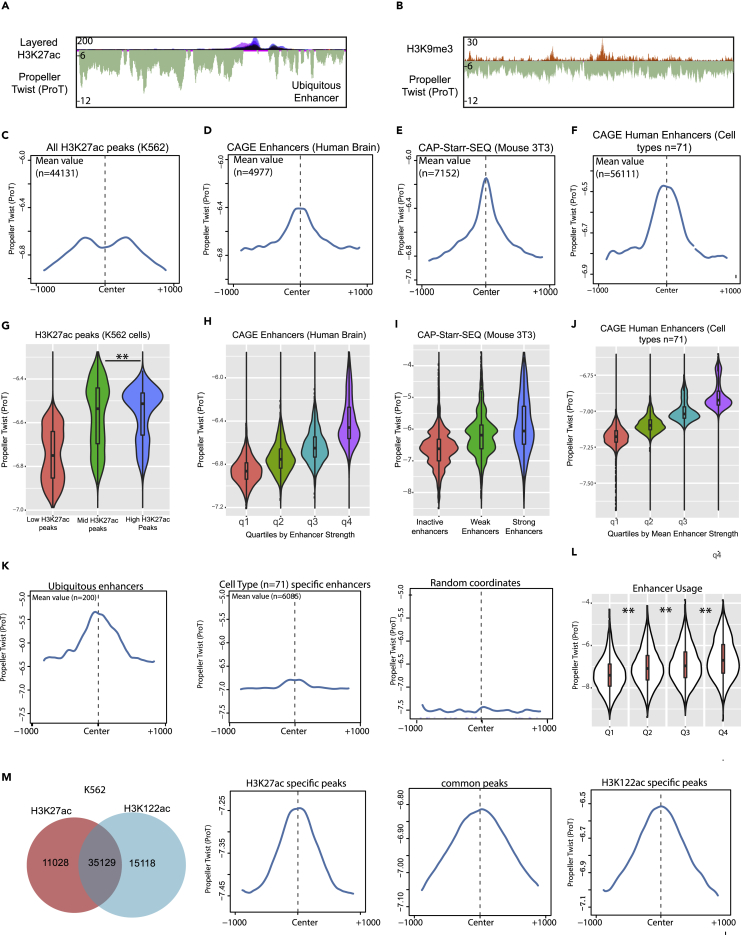
Propeller Twist Encodes the Regulatory Potential of Genetic Elements (A) UCSC genome browser track displaying layered H3K27ac tracks and Propeller Twist (ProT) showing higher ProT values at H3K27ac positive regions. (B) UCSC genome browser track displaying H3K9me3 (K562) tracks and ProT showing lower ProT values at H3K27me3 positive regions. (C) Density plot depicting ProT at H3K27ac peaks identified in K562 cells. (D–F) Same as (C) but as expressed enhancers in Human Brain identified in CAGE experiments (D), STARRseq identified enhancers in mouse NIH3T3cells (E) and all CAGE defined enhancers across 71 cell types from FANTOM5 atlas (F). (G) Violin-boxplot depicting ProT levels at H3K27ac peaks clustered into three categories based on enrichment (low, mid, and high). (H) Same as G but at four groups obtained from increasing quartile expression of Human Brain enhancers identified from CAGE experiments. (I) Violin-boxplot depicting ProT levels at capSTARR-seq defined enhancers classified into three classes as inactive, weak, and strong enhancers. (J) Same as H but at quartiles defined by mean expression across 71 cell types. (K) Density plot depicting ProT profiles at genomic coordinates marked by ubiquitous enhancers (left), cell-type-specific enhancers (mid) and random coordinates (right). (L) Violin-boxplot depicting ProT levels at enhancers ranked into four quartile groups into the increasing order of enhancer usage (cross-cell type usage of enhancers). (M) Venn-diagram depicting overlap of peaks from H3K27ac and H3K122ac ChIP-seq study in Human K562 cells (left). ProT profile plotted as density plot across H3K27ac specific peaks identified from this comparison (second from left), common peaks (second from right), and H3K122ac (right). See also Figure S3.

Intrigued by the above findings, we next attempted to perform a detailed characterization of high ProT regions. In line with our previous findings, we find that “high ProT” sequences are prevalent at regions enriched with H3K27ac, a marker of active promoter and enhancer regions, whereas they are depleted at regions enriched in the repressive epigenetic mark H3K9me3 (Figures 3A and 3B). To further validate these findings, we segmented the epigenome of human K562 myeloma cells into 15 different chromatin states using ChromHMM (Ernst and Kellis, 2012) and determined their ProT levels. Consistent with the previous observations, we found generally higher ProT levels at genomic regions marked by active chromatin marks as compared with repressive ones (Figures S3A–S3C). An interesting exception was H3K27me3, a repressive mark, which correlates with higher ProT levels (Figure S3C). This may be explained by the fact that H3K27me3 marks certain genomic regions that permit enhancer activity under certain physiological conditions (Taberlay et al., 2011). Furthermore, this mark is also known to be present at “poised” promoters that represent a transcription ready state (Bernstein et al., 2006).

We next extracted experimentally validated regulatory regions from a variety of cell types and analyzed their ProT profiles. Strikingly, we noticed that the regulatory elements defined by H3K27ac mark show a highly characteristic distribution of ProT levels where ProT peaks appear symmetrically next to the center of H3K27ac peaks (Figure 3C). Furthermore, ProT peaks overlap with the centers of regulatory elements identified by CAGE or STARR-seq experiments, suggesting that ProT is an intrinsic property of regulatory regions (Figures 3D and 3E). Extended analysis of CAGE-defined enhancers across 71 cell types further supports these findings (Andersson et al., 2014) (Figures 3F and S3D).

Next, we sought to monitor the correlation of ProT levels with enhancer activity in a quantitative manner. Because H3K27ac levels at enhancers are known to correlate with gene expression levels, we used this as a proxy for enhancer usage (Karlic et al., 2010). A comparison of H3K27ac enrichment with ProT levels demonstrated a clear relationship (Figure 3G), which was also true with CAGE- or STARR-seq-determined enhancer strength across multiple cell types (Figures 3H–3J). Based on these observations we also hypothesized whether ProT levels could also help discriminate enhancer usage across cell types. Interestingly, we indeed observed that higher ProT level-containing regions tend to be ubiquitous enhancers, whereas those showing lower ProT level were enhancers of cell-type specific genes (Figure 3K). This may relate to an easy activatable state of housekeeping genes versus those of cell-type specific genes that generally require distinct machinery and program to induce their expression. Further, we observed that the ProT levels correlate with expression levels (Figures 3L and S3E), suggesting that the transcriptional competence is potentially orchestrated at the genetic level by DNA shape features.

The current repertoire of histone modifications does not seem sufficient to define all genomic regulatory elements, and efforts are continuously being made to uncover new chromatin features that allow mapping all enhancers. In line with this, H3K122ac modification was shown to mark enhancers that do not exhibit any H3K27ac mark (Pradeepa et al., 2016). Further corroborating our previous observations, H3K122ac positive and H3K27ac negative enhancers show a characteristic high ProT profile (Figure 3M). Thus, high ProT levels constitute a common feature of enhancers irrespective of the chromatin mark defining these regions. These findings argue that high ProT levels constitute a common feature of enhancers, which overrides limitations of other modes of predicting enhancers including those based on the epigenetic state.

### ProT Profile Is a Deterministic Feature of Enhancers

**Figure 4:**
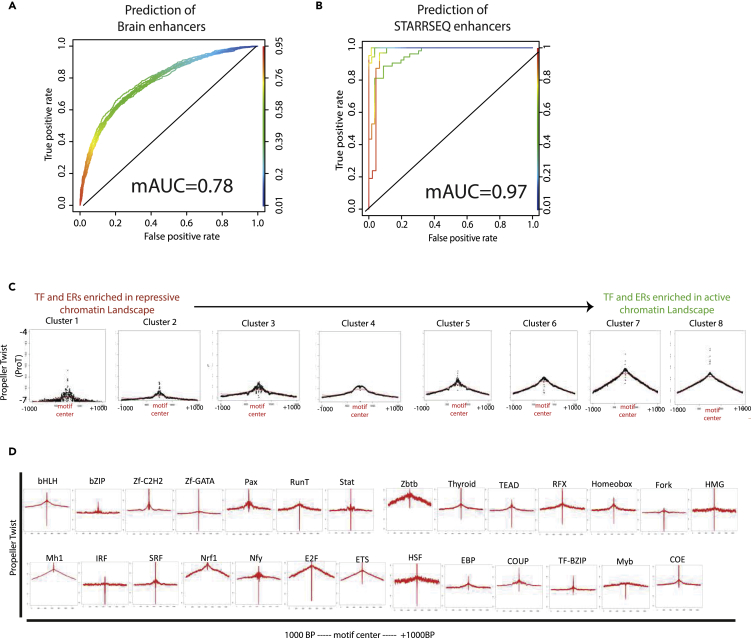
Propeller Twist Is Deterministic of Regulatory Potential of Genetic Elements (A) Receiver operator curve (ROC) depicting prediction accuracy of SVM models (n = 9) trained to classify between enhancer sequences in brain and random genome loci using 2000BP single nucleotide ProT predictions. (B) Same as A but SVM models (n = 5) trained at capSTARR-seq defined enhances in mouse NIH3T3cells. (C) ProT density plots over each of the clusters (identified in Figure S4A) arranged from left to right in the increasing order of enrichment in repressive to active chromatin landscape. (D) ProT density plots as median over all factors across particular transcription factor family. See also Figure S4.

To further establish predictive nature of ProT levels in priming genomic regions for a gene regulatory function we developed SVM (Support Vector Machine) models to classify between random genomic loci and CAGE-defined brain enhancers using single nucleotide ProT values across a 2000 bp window. The resulting nine models could classify the location of enhancers at randomly chosen genomic sites with very high accuracy (mAUC = 0.78) (Figure 4A). Next, we trained five models in a similar manner to identify STARR-seq defined enhancers from mouse NIH3T3cells. Again, our SVM models closely predicted enhancer locations (AUC = 0.96) (Figure 4B).

Enhancers are known to contain multiple TF (Transcription Factor) binding sites, and given a strong relationship between ProT and enhancer occurrence and usage, we next had a closer look at TF motifs at ProT profiles. Here we clustered TF-bound motifs, as derived from actual ChIP-seq assays for these TFs, into eight different clusters based on various histone modification patterns at these sites as hallmark of euchromatin and heterochromatin (Figures S4A–S4C). Interestingly, an analysis of ProT levels at the center of motif at these TF bound sites for each of these clusters showed that the activator and repressor TF motifs can be clearly delineated by ProT levels. The TFs that function primarily as activators preferably target motifs embedded in high ProT environments, whereas TFs acting mainly as repressors bind motifs within lower ProT environments (Figures 4C and 4D). These findings argue that ProT profile is a deterministic feature of enhancers and can predict the active distal gene regulatory landscape with high accuracy.

### Cell-Fate Switches Involve a Transient Usage of Low ProT Regulatory Elements

**Figure 5:**
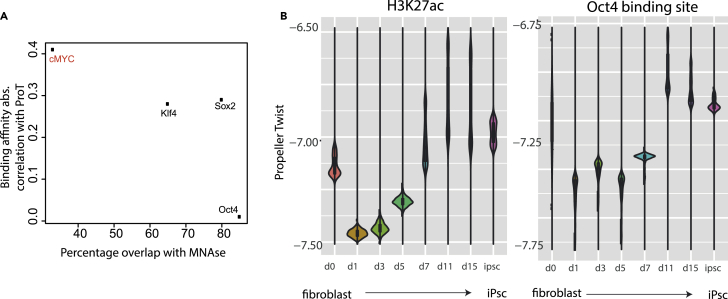
Switch between Distinct Cell-Fates Involves Transient Usage of Low ProT Regulatory Elements (A) Scatter plot with enrichment of reprogramming factors on nucleosome (stage; fibroblasts) on X axis as determined by the study by Soufi et al. (Soufi et al., 2015) and binding affinity (stage: 48 h of reprogramming) correlation with ProT on Y axis. (B) Violin-boxplots with ProT levels at H3K27ac peaks (left) and Oct4 binding sites (right) at various stages of reprogramming on mouse fibroblasts into iPSC cells.

We next explored the ProT dependency landscape of different TFs to reveal the possible impact of DNA structure on the function of general vs cell-fate-determining TFs. Toward this, we looked for systems that involve dynamic reprogramming of cell-fate using defined TFs. Somatic cells can be efficiently reprogrammed into an embryonic stem cell state, i.e. induced pluripotent stem cells (iPSCs), using a distinct set of TFs, namely Oct4, Sox2, Klf4, and c-Myc (Takahashi and Yamanaka, 2006). Therefore, using datasets from a previous study we analyzed binding of these four TFs during fibroblast-to-iPSC reprogramming and assessed its relation to ProT levels and nucleosome occupancy in pre-induced human fibroblasts, as measured by MNase sequencing (MNase-seq) (Chronis et al., 2017). Interestingly, c-Myc showed lesser affinity for nucleosome bound regions, whereas Oct4, Sox2, and Klf4 preferentially targeted nucleosome-enriched sites (Figure 5A). These data are consistent with a previous finding that Oct4, Sox2, and Klf4, but not c-Myc, could function as pioneer TFs during reprogramming by virtue of their ability to target “closed” chromatin sites (Soufi et al., 2015). Furthermore, while c-Myc, Klf4 and Sox2 ChIP-seq enrichment relied on levels of ProT, this was less so in the case of Oct4.

Intrigued by these findings, we analyzed the rewiring of the active chromatin landscape using H3K27ac mark and its relationship with Oct4 binding dynamics during distinct stages of reprogramming. Strikingly, although H3K27ac sites in either fibroblasts or iPSCs show stable ProT levels, those occurring during any of the transient states during reprogramming show a significant drop in their ProT levels (Figure 5B). This pattern was closely mimicked by genomic regions targeted by Oct4 at distinct stages of reprogramming (Figure 5B). These results suggest that while Oct4 binding and enhancer activation occurs at “low ProT” regions in transient cell states occurring during reprogramming, the acquisition of a fully reprogrammed cell-fate involves utilization of “average ProT” sites as enhancers (Figure 5B). Altogether, these findings imply that although high ProT sites are hallmark of enhancers in defined cell types, relatively lower ProT sites may play a crucial role during setting up of these cell-fates during reprogramming and potentially in development.

### ProT Levels Correlate with Occurrence and Phenotypic Consequences of Mutations

**Figure 6:**
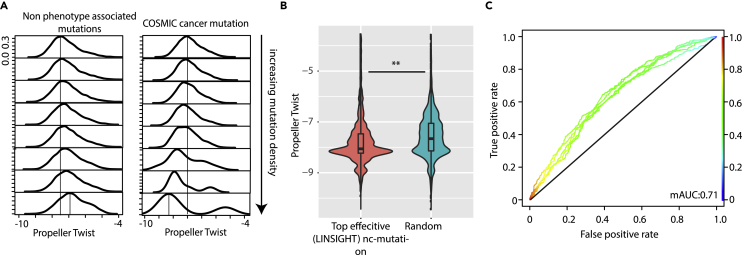
Functional Implications of Differential ProT Levels in the Genome (A) Left: density plot depicting ProT density across eight categories of genomic loci (1000 bp) classified into increasing mutation density of non-phenotype associated mutation class. Right: same as Left, but for mutations associated to cancer in COSMIC database (Forbes et al., 2017). (B) Violin-boxplot for effectivity of genomic loci to be effective in terms of phenotype as determined by LINSIGHT database (Huang et al., 2017) in high and low ProT classes. (C) SVM models accuracy plot as ROC curve depicting efficient classification of genomic classes as effective or otherwise as determined LINSIGHT. See also Figure S5.

Given our findings of a deterministic role of ProT in the regulatory potential of distinct genomic sites, we next assessed the differential sensitivity of ProT sites to tolerate mutations. Strikingly, our analysis revealed a higher occurrence of random mutations (i.e. non-phenotype associated) at “high ProT” regions of the genome (Figures 6A and S5). This implies that inherent higher DNA accessibility plays a critical role in enhancing mutability, possibly because mutagenic agents or machinery have an easier access to such sites. In contrast, the occurrence of cancer-associated mutations (i.e. phenotype associated) is higher in genomic loci characterized by a lower ProT (Figures 6A and S5). It is likely that these low ProT sites offer reduced access to DNA repair machineries and consequently more likely to result in phenotypic consequences.

We further employed LINSIGHT predictions to determine effectivity of SNPs in causing a detectable phenotype (Huang et al., 2017). We analyzed genomic segments in 1 kb bins and found that ProT levels are lower at phenotype-associated SNPs than at random genomic loci, in line with our previous observations (Figure 6B). We further implemented SVM models trained on ProT information for 2 kbp genomic segments to classify effective versus random genomic loci in terms of phenotypic association. Strikingly, the generated models were able to predict whether mutation at a specific locus could be phenotypic with a considerably high accuracy (Figure 6C). Taken together, we show that ProT levels are correlated with occurrence and phenotypic consequences of mutations.

## Discussion

The DNA sequence composition is known to influence local DNA shape across the genome (Parker et al., 2009). However, at a broader scale, the DNA sequence and structures appear as independent and deconvolved features (Abe et al., 2015). Previous studies have hinted upon a role of DNA sequence as well as topography in determining certain epigenetic features (Arnold et al., 2017, Parker et al., 2009, Rusk, 2014, Wang and Moazed, 2017). The genetic features were also shown to be important in determining TF access on the DNA (Yang et al., 2017b, Zhou et al., 2015). However, whether the local DNA biophysical features play any role in a chromatin context and in gene regulation is unknown. This study provides unprecedented insights into the deterministic role of DNA biophysical features in governing epigenetic and gene regulatory landscape underlying cell identity and function.

Our study has discovered ProT as a novel proxy for measuring inherent surface accessibility as determined by OH-radical cleavage mapping and local DNA flexibility as probed by atomic force microscopy. Further assessment of distribution of different ProT sequences within the 3D nuclear space revealed that higher ProT regions are enriched in euchromatic domains and are more interiorly located in 3D genome structure. In contrast, lower ProT regions are enriched in heterochromatic domains and are more exterior in their location within the 3D genome structure. These intriguing results implied a novel role for ProT levels of DNA sequences in guiding DNA surface accessibility and flexibility, epigenetic state, and ultimately the nuclear organization of distinct chromatin domains. As LADs are known to be AT-rich, future studies should attempt to dissect any contribution of DNA sequence versus DNA shape to our observations (van Steensel and Belmont, 2017). Importantly further, although our analysis found ProT to positively correlate with the DNA surface accessibility, HelT showed negative correlation. Further investigation is required to uncover the relevance of this anti-correlation, in particular towards the gene regulatory landscape.

A large scale, systematic analysis showed specific enrichment of ProT at the regulatory elements that were previously identified by a number of independent experimental measures in multiple cell-types and across species. Importantly, further, the regulatory potential of the genomic regions showed a strong correlation with ProT levels. Additional analysis revealed that ProT is a deterministic feature of the enhancers irrespective of the chromatin mark used for their identification. Strikingly, the predictive power of ProT to identify sequence-intrinsic enhancer features, as experimentally measured by STARR-seq (Arnold et al., 2013, Muerdter et al., 2015), was very high, suggesting that ProT predictions are able to decode sequence logic with confidence in such experiments and offers an alternative to heavy experimentation-based analysis. Collectively, our findings argue that the local DNA biophysical features hold the potential to prime a genomic region for particular epigenetic state and gene regulatory potential, thus revealing an underestimated role of DNA structure in guiding genome function. This is further in line with previous studies that have shown that the DNAshape algorithm works better than certain k-mer (2,3) combinations for some biological functions (Abe et al., 2015, Mathelier et al., 2016).

ProT, along with other DNA shape features, have been shown to contribute to TF access DNA (Yang et al., 2017b, Zhou et al., 2015). Our analysis shows that the activator and repressor TFs have an inverse binding affinity with ProT levels. Importantly, further, in contrast to general TFs, pioneer TFs have lesser dependency on these DNA features for their binding. A number of efficient reprogramming TFs are known to be pioneer TFs (Pataskar et al., 2016, Soufi et al., 2015). Interestingly, employing pioneer TF binding and H3K27ac data during distinct stages of cellular reprogramming of a differentiated to a pluripotent state, we find that the switch between cell-fates involves a transient usage of low ProT as regulatory elements. In further support of this highly influential role of ProT in guiding gene regulation, DNA flexibility, and surface accessibility, we found a higher occurrence of random or non-phenotype-associated mutations at “high ProT” regions of the genome, whereas the occurrence of phenotype-associated mutation is higher at lower ProT genomic regions, which we identified to mark cell-identity enhancers. This implies that high ProT-imposed increased DNA accessibility plays a critical role in enhancing mutability in the genome, potentially because mutagenic agents or machinery have an easier access to such sites. A previous study has suggested that the GC-rich sequences tend to have less pronounced ProT values, whereas AT-rich sequences tend to have more negative ProT values (Hancock et al., 2013). It is thus also likely that the observed relationship to the mutation rate is also influenced to certain extent by the sequence composition and warrants further investigation.

Altogether, our work provides unprecedented insights into the gene regulatory landscape encoded in the DNA biophysical features. Our findings open a new area of investigation that was previously underestimated for its relevance and vouches for the necessity to include DNA shape features while studying epigenetic gene regulatory mechanisms in various contexts. Our study hypothesizes that DNA sequences have evolved in a highly orchestral manner, wherein genomic DNA is segmented into compartments of different inherent biophysical states, which are then chosen to be in different nuclear chromatin compartments of different regulatory potential. Follow-up studies should aim to investigate how epigenetic machineries such as DNA modifying enzymes alter the DNA structure at specific sites. Furthermore, it will also be important to determine how combinatorial TF binding influences DNA flexibility and if this crosstalk is relevant for cell-fate decisions during development. In addition, it will be interesting to investigate how genomic sites with various ProT levels are utilized in the 3D nuclear space in response to external cues during development and in various diseases. Such investigations will ultimately decode the relationship between genetic and epigenetic mechanisms, which is key for a comprehensive understanding of genome function in health and disease.

### Limitations of the Study

DNAshape algorithm predicts shape from DNA sequence, and hence it will show high correlation to the sequence. Given this close dependency, it is almost impossible to fully dissect the contribution of DNA sequence versus shape. This limitation is valid for several previous publications using DNAshape algorithm as well as our own study.

## Methods

All methods can be found in the accompanying Transparent Methods supplemental file.

## Supplemental Information

Document S1. Transparent Methods and Figures S1–S5

Data S1. Details on DNA Sequences Analyzed by AFM, Related to Figures 1 and S1
