# Supplementary material for: Deciphering the Gene Regulatory Landscape Encoded in DNA Biophysical Features
Source: iScience. 2019 Oct 31;21:638–49. doi: 10.1016/j.isci.2019.10.055 (PMC6889597; doi:10.1016/j.isci.2019.10.055)
Supplement: Document S1. Transparent Methods and Figures S1–S5 [file mmc1.pdf]

ISCI, Volume *21*

Supplemental Information

Deciphering the Gene Regulatory Landscape Encoded in DNA Biophysical Features

Abhijeet Pataskar, Willem Vanderlinden, Johannes Emmerig, Aditi Singh, Jan Lipfert, and Vijay K. Tiwari

SUPPLEMENTARY FIGURE LEGENDS

Supplementary Figure 1: Related to Figure 1.

(A-C) Genome-wide correlations of DNA shape features; Major Groove Width (MGW) and Roll (A), MGW and Propeller Twist (ProT,B) and ProT and Roll (C) (D) Left: Measurements of Contour length distributions; schema (top) and Fitted regression line of contour length distributions (below) Mid: Density plot of contour length distribution in low ProT control (above) and high ProT construct (below). Right: Schema for measurement of contour length with length scales (5nm).

Figure S1

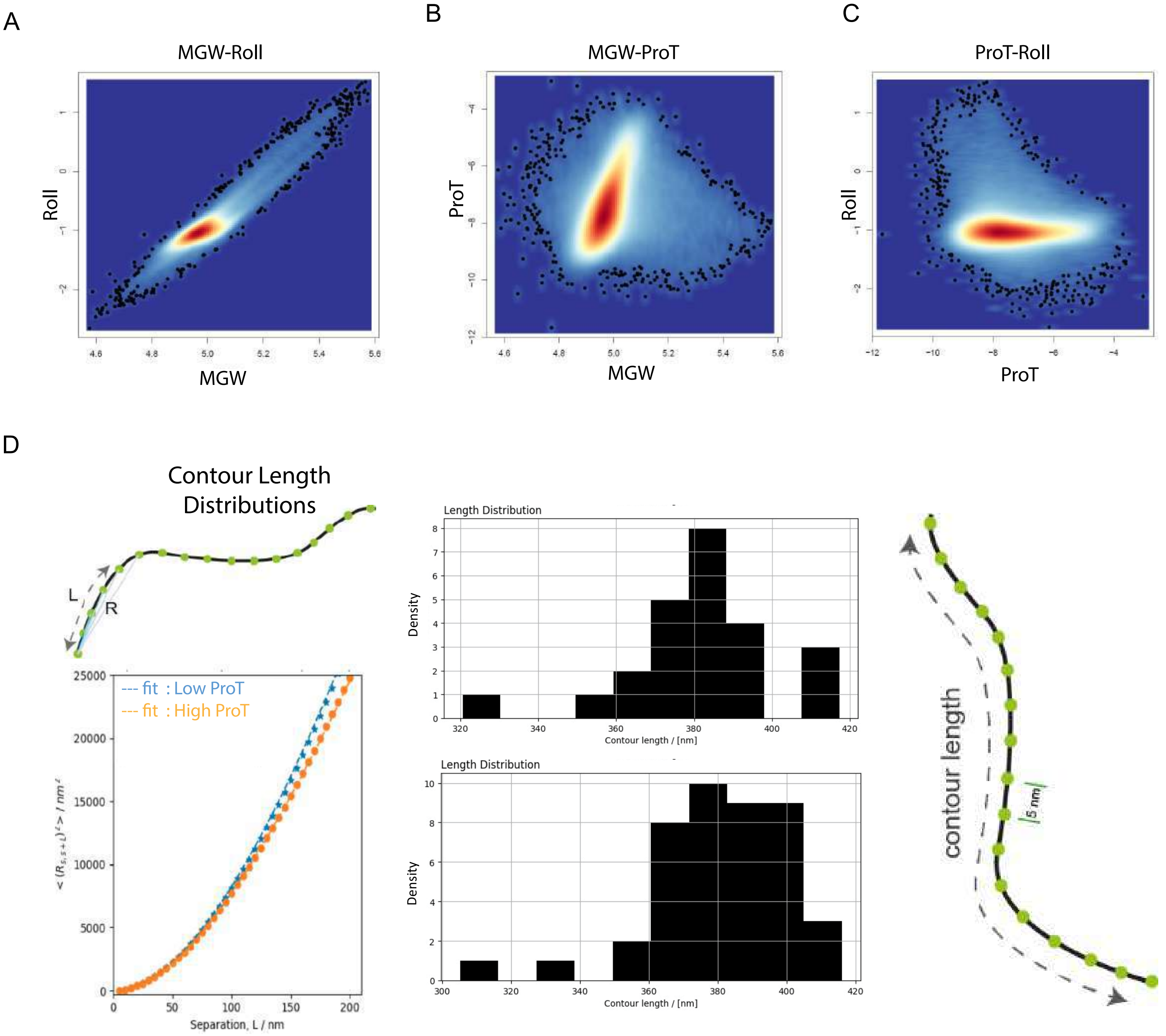
A
B
C
MGW-Roll
MGW-ProT
ProT-Roll
Roll
ProT
MGW
D
Contour Length Distributions
L
R
--- fit : Low ProT
--- fit : High ProT
Separation, L / nm
Length Distribution
Density
Contour length / [nm]
contour length
5 nm

Supplementary Figure 2: Related to Figure 2.

(A) Genome Surface maps highlighted with Euchromatin in blue (above) and Heterochromatin in red (below) derived from the reconstructed genome structure from the single cell HiC experiments in mES cells. (B) Correlation scatter matrix with correlation coefficients (numbers) derived genome-wide from the information of surface depths in all analyzed 7 cells, ProT, Euchromatin and Heterochromatin density. (C) Cross correlation scatter plot from the surface depths of genomic loci from seven different reconstructed single cell genome structures of mES cells (D) Line plot depicting linear profile of surface depths (black) and ProT (red) across lengths of each chromosomes in mouse ES cells.

Figure S2

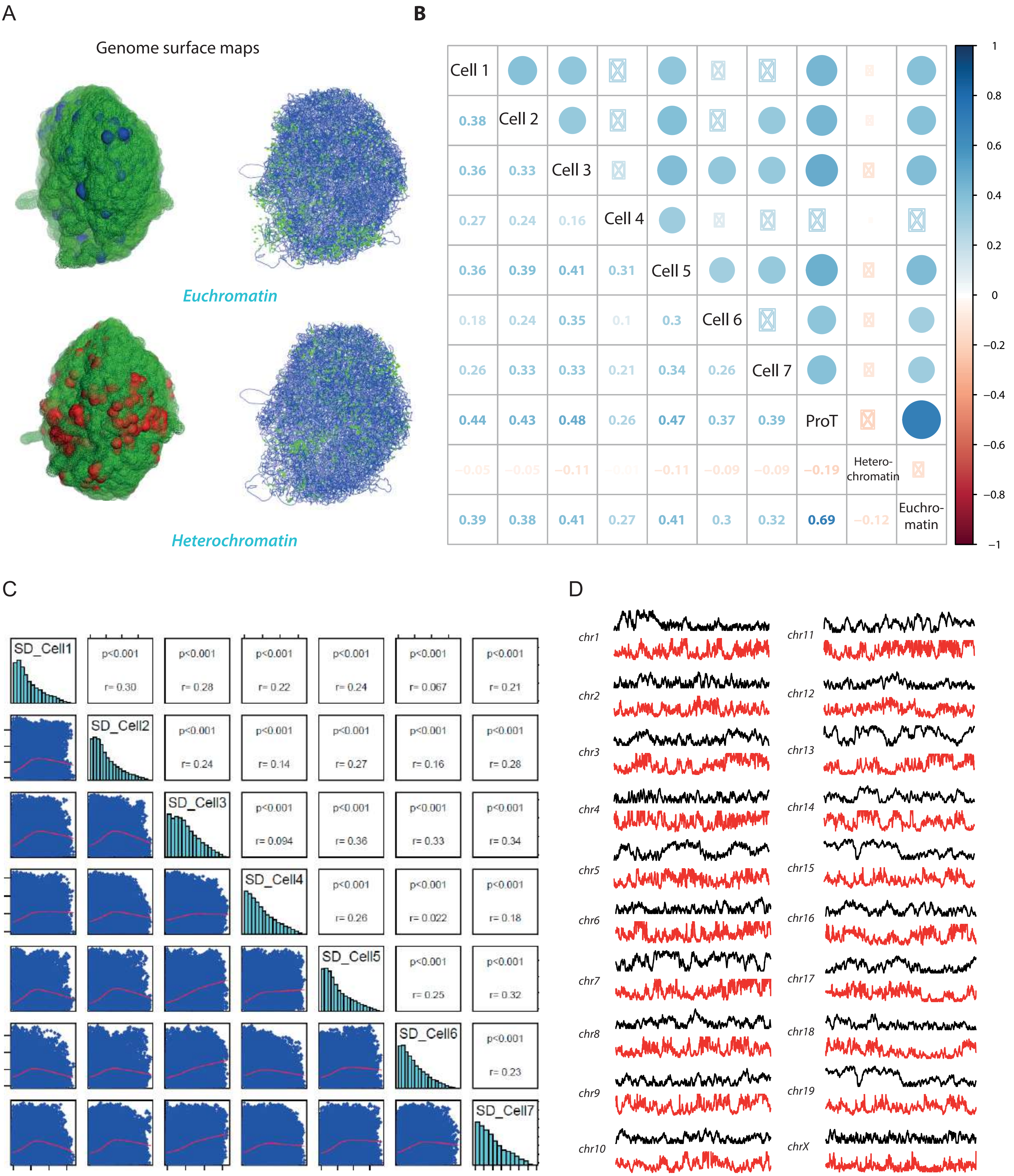
A
Genome surface maps
Euchromatin
Heterochromatin
B
Cell 1
0.38 Cell 2
0.36 0.33 Cell 3
0.27 0.24 0.16 Cell 4
0.36 0.39 0.41 0.31 Cell 5
0.18 0.24 0.35 0.1 0.3 Cell 6
0.26 0.33 0.33 0.21 0.34 0.26 Cell 7
0.44 0.43 0.48 0.26 0.47 0.37 0.39 ProT
−0.05 −0.05 −0.11 −0.01 −0.11 −0.09 −0.09 −0.19 Hetero-chromatin
0.39 0.38 0.41 0.27 0.41 0.3 0.32 0.69 −0.12 Euchro-matin
1 0.8 0.6 0.4 0.2 0 −0.2 −0.4 −0.6 −0.8 −1
C
SD_Cell1
p<0.001 r= 0.30
p<0.001 r= 0.28
p<0.001 r= 0.22
p<0.001 r= 0.24
p<0.001 r= 0.067
p<0.001 r= 0.21
SD_Cell2
p<0.001 r= 0.24
p<0.001 r= 0.14
p<0.001 r= 0.27
p<0.001 r= 0.16
p<0.001 r= 0.28
SD_Cell3
p<0.001 r= 0.094
p<0.001 r= 0.36
p<0.001 r= 0.33
p<0.001 r= 0.34
SD_Cell4
p<0.001 r= 0.26
p<0.001 r= 0.022
p<0.001 r= 0.18
SD_Cell5
p<0.001 r= 0.25
p<0.001 r= 0.32
SD_Cell6
p<0.001 r= 0.23
SD_Cell7
D
chr1
chr2
chr3
chr4
chr5
chr6
chr7
chr8
chr9
chr10
chr11
chr12
chr13
chr14
chr15
chr16
chr17
chr18
chr19
chrX

Supplementary Figure 3: Related to Figure 3.

(A) ChromHMM based clustering of genome into segments with 15 different chromatin states. (B) Propeller Twist (ProT) levels depicted as box-plots in each of these 15 different chromatin states. Highlights: red- heterochromatin, green-euchromatin. (C) Line plot depicting genome-wide correlation coefficients of ProT with different chromatin marks. (D) Above: Example scatter plots from two cell types with enhancer strength as measured by CAGE experiment on X-axis and ProT on y-axis. Below: Same information depicted as regression line plot for ProT when enhancers are arranged in increasing order of strength. (E) ProT levels plotted as density plot to -50 KB to +50 KB of TSS and TES respectively of genes classified into four quartiles by expression value in Human myeloma K562 cells.

Figure S3

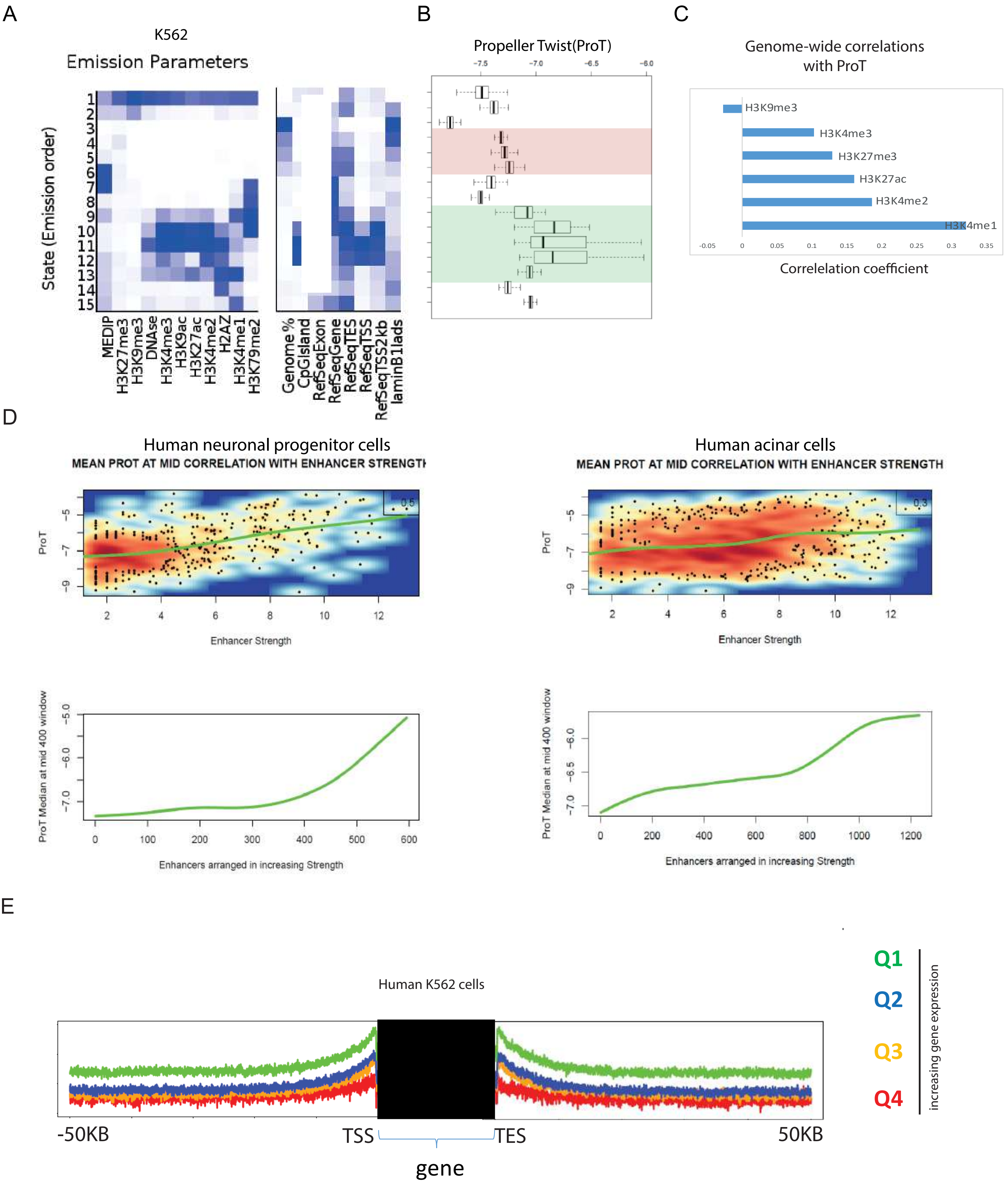
A
K562
Emission Parameters
State (Emission order)
1
2
3
4
5
6
7
8
9
10
11
12
13
14
15
MEDIP
H3K27me3
H3K9me3
DNAse
H3K4me3
H3K9ac
H3K27ac
H3K4me2
H2AZ
H3K4me1
H3K79me2
Genome %
CpGIsland
RefSeqExon
RefSeqGene
RefSeqTES
RefSeqTSS
RefSeqTSS2kb
laminB1lads
B
Propeller Twist(ProT)
-7.5
-7.0
-6.5
-6.0
C
Genome-wide correlations with ProT
H3K9me3
H3K4me3
H3K27me3
H3K27ac
H3K4me2
H3K4me1
-0.05
0
0.05
0.1
0.15
0.2
0.25
0.3
0.35
Correlelation coefficient
D
Human neuronal progenitor cells
MEAN PROT AT MID CORRELATION WITH ENHANCER STRENGTH
0.5
ProT
Enhancer Strength
ProT Median at mid 400 window
Enhancers arranged in increasing Strength
Human acinar cells
MEAN PROT AT MID CORRELATION WITH ENHANCER STRENGTH
0.3
ProT
Enhancer Strength
ProT Median at mid 400 window
Enhancers arranged in increasing Strength
E
Human K562 cells
-50KB
TSS
TES
50KB
gene
Q1
Q2
Q3
Q4
increasing gene expression

Supplementary Figure 4: Related to Figure 4.

(A) PCA plot depicted distribution of Transcription factor binding enrichment over chromatin marks, thereby clustered into 8 different groups colored differently. Vectors represent the contributing features for the PCA distribution. PCA overall classifies transcription factors on PC1 with enrichment of factors on repressive chromatin landscape on X-axis and on active chromatin landscape on Y-axis. Data acquired from K562 cells. (B-C) Example of cluster 8 (B) and cluster 1 (C) transcription factors where enrichment of different chromatin marks.

Figure S4

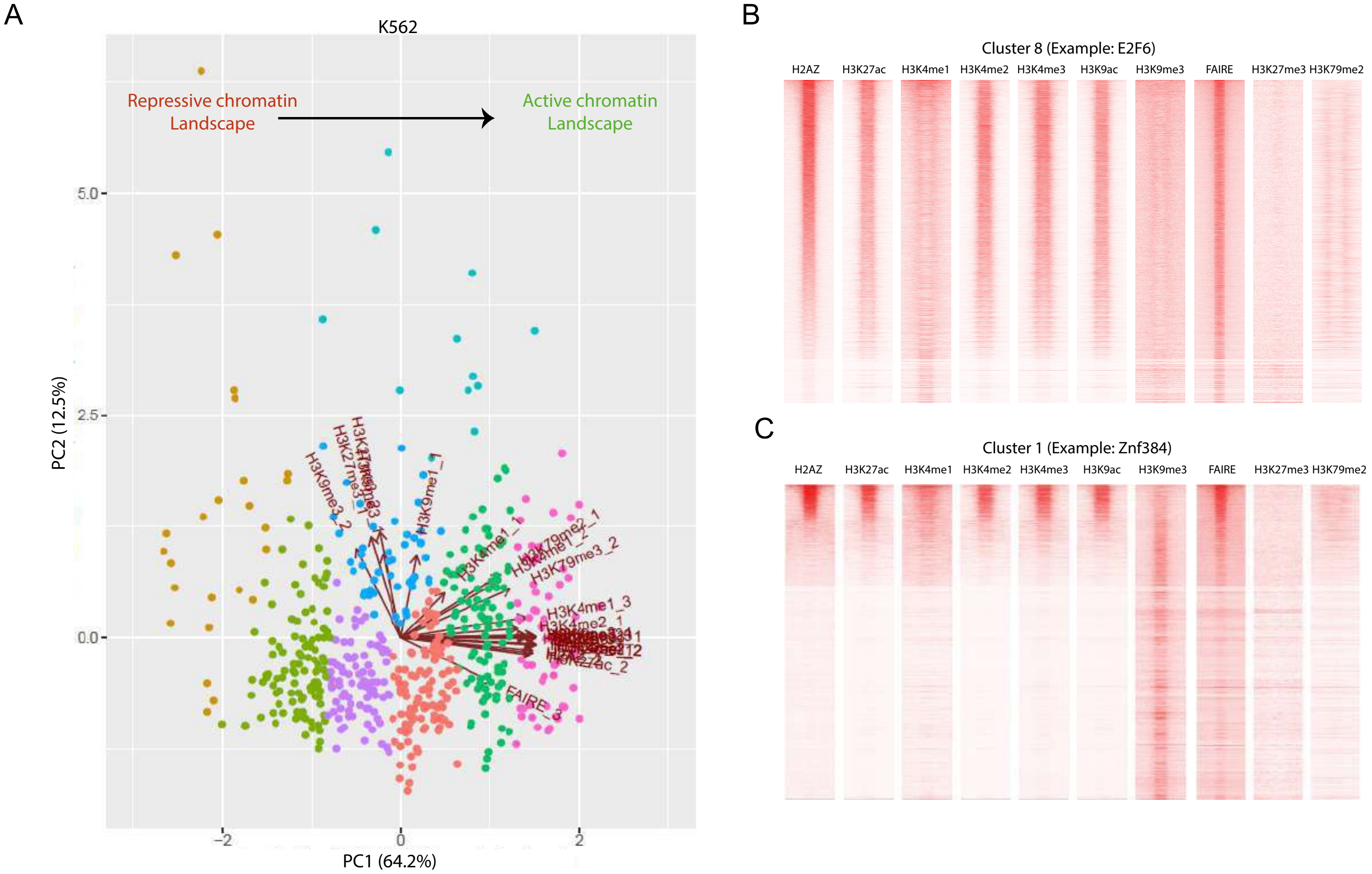
A
K562
Repressive chromatin Landscape
Active chromatin Landscape
PC2 (12.5%)
PC1 (64.2%)
5.0
2.5
0.0
-2
0
2
H3K9me3_2
H3K27me3_1
H3K9me1_
H3K4me1_1
H3K79me2_1
H3K79me3_2
H3K4me1_3
H3K4me2_1
FAIRE_3
B
Cluster 8 (Example: E2F6)
H2AZ
H3K27ac
H3K4me1
H3K4me2
H3K4me3
H3K9ac
H3K9me3
FAIRE
H3K27me3
H3K79me2
C
Cluster 1 (Example: Znf384)
H2AZ
H3K27ac
H3K4me1
H3K4me2
H3K4me3
H3K9ac
H3K9me3
FAIRE
H3K27me3
H3K79me2

Supplementary Figure 5: Related to Figure 6.

Violin-box plots depicting ProT levels at distinct enhancer regions arranged in the increasing order of occurrence of phenotypic (COSMIC) mutations.

Figure S5

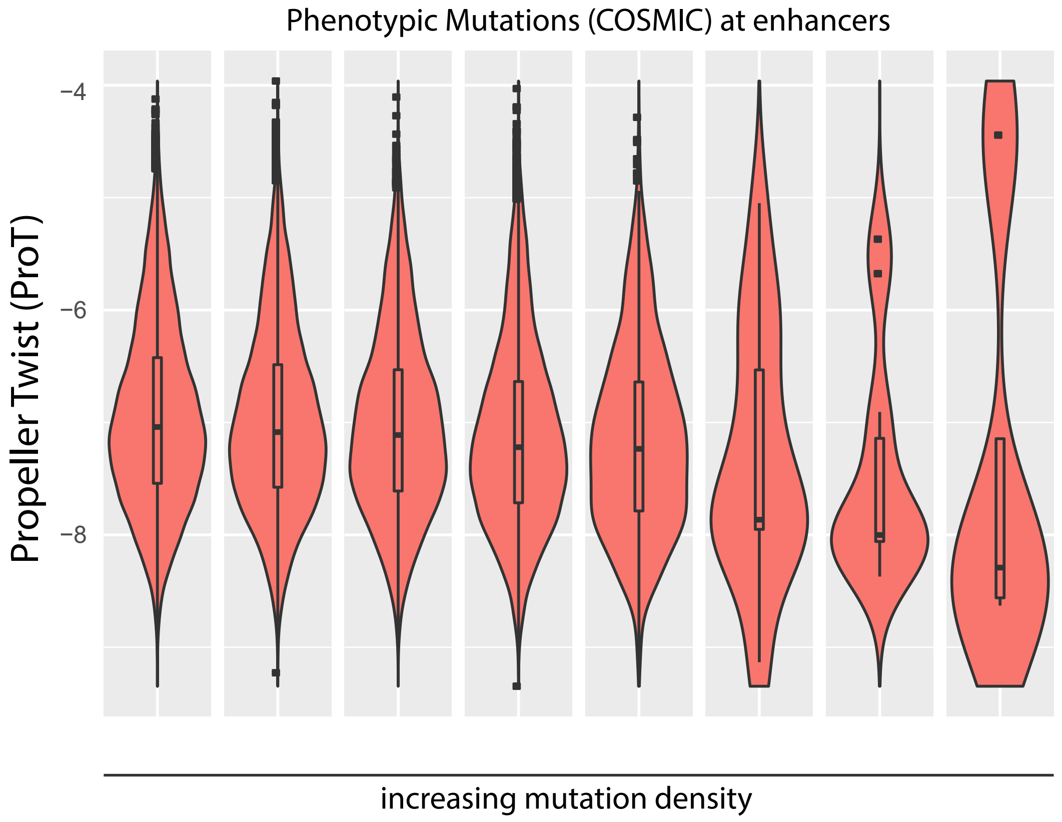
Phenotypic Mutations (COSMIC) at enhancers
Propeller Twist (ProT)
−4
−6
−8
increasing mutation density

TRANSPARENT METHODS

Preparation of DNA constructs for AFM imaging

DNA sequences (1167 bp long) were generated by polymerase chain reaction using Phusion master mix (2x Phusion High-Fidelity PCR Master Mix, Thermo Fisher Scientific Inc., Waltham, MA, USA) using plasmid pUC57 with sequences of interest introduced at HindIII site (biocat) as a template and using the sequences CCC AGT CAC GAC GTT GTA AAA CG and AGC GGA TAA CAA TTT CAC ACA GG as forward and reverse primers respectively. PCR products were purified using a PCR cleanupCleanup kit and stored in Tris-EDTA buffer (10 mM Tris- HCl, pH = 8.0; 1 mM EDTA) at 4° C. To verify successful amplification of the desired sequence, the product was analyzed on a 1 % (w/v) agarose gel.

AFM imaging

A buffer solution (12 mM $MgCl_2$; 10 mM Tris-HCl, pH = 8.0) containing 1167 bp linear DNA construct at 0.5 ng/ L was deposed onto freshly cleaved mica by drop-casting for 30 seconds, followed by gentle rinsing with milliQ water (25 mL) and drying under N^2. AFM imaging was performed on a Multimode AFM equipped with a Nanoscope III controller (Digital Instruments) and a type E scanner (Bruker). Images were recorded on dried samples, under ambient conditions, and using silicon cantilevers (Nanosensor; SSS-NCHR; resonance frequency ≈ 300 kHz). Typical scans were recorded at 1–3 Hz line frequency, with optimized feedback parameters and 512 × 512 pixels of 1.9 nm each.

AFM data analysis

AFM topography images were loaded in Scanning Probe Imaging Processor software (v6.4) and background corrected using global fitting with a second order polynomial and in a line-by-line fashion using the histogram alignment routine. The processed images were saved in ASCII format and analyzed using custom-written code implemented in Python framework. Image analysis involves finding (x,y) coordinates that define the DNA curvilinear length by tracing the molecular contour with a step-length l = 5 nm, following the routine introduced by Wiggins et al. (Wiggins et al., 2006). Bend angles are defined as the deviation from linearity between a certain set of tangent vectors separated by l. In total, we traced 87168 bend angles from 1226 imaged DNA molecules. The energy landscape for bending was reconstructed from the corresponding bend angle distribution by taking the negative logarithm. The energy required to introduce a bend increases with the bend angle θ. Up to θ ~ 1 rad the increase is approximately quadratic and in agreement with the prediction of the worm-like chain model $E_{WLC}(\theta) = 1/2 \cdot k_B T \cdot (P/l) \cdot \theta^2$, with P ~ 55 nm (Figure 1C, dashed line), where k_B is the Boltzmann constant, T the absolute temperature (295 K), and l the segment

length (5 nm in our analysis). For bending angles θ > 1 rad, the energy to bend the DNA grows more slowly -in other words large bends occur more frequently- than predicted by the WLC model, as has been reported previously (Wiggins et al., 2006). While both the control and high ProT sequences deviate from the WLC prediction, the energy required to introduce a large bend for the high ProT sequences is lower and, therefore, deviates more strongly from the WLC prediction compared to the control sequences.

Further, the (x,y) coordinates are used to quantify the end-to-end distances R between any two traced positions as a function of their separation L along the contour. In turn, the relation between the mean squared end-to-end distance $\langle R^2 \rangle$ and L can be used to examine surface equilibration using the formula $\langle R^2 \rangle = 4\, P\, L\, (1 - 2\, P/L\, (1 - \exp(-L / 2P)))$ with P the bending persistence length.

DNAshape and OH-radical cleavage prediction

DNAshapeR package (Chiu et al., 2016) was used to generate and plot DNA shape feature predictions, namely Propeller Twist, Major Groove Width, Helix Turn and Roll. Density plots of these features were plotted using DNAShapeR (Chiu et al., 2016). OH-radical cleavage predictions were obtained from ORCHID2 (Greenbaum et al., 2007) server. For genome-wide predictions of DNAshape features, bigwig files (mm9 and hg19 for mouse and human respectively) were downloaded from GBShape (Chiu et al., 2015). Correlation within DNAshape (Chiu et al., 2015) features and with OH-radical cleavage intensity (ORCHID) were done by using Deeptools (Ramirez et al., 2014) at the resolution of 1KB.

Reconstruction of 3D Genome Structures and overlay with Chromatin features and Propeller Twist predictions

HDF5 Datafiles containing genome structure features reconstructed from single-cell HiC experiments of seven mouse ES cells were downloaded from supplementary information provided in study from Stevens et al (Stevens et al., 2017). The surface depths with resolution of 1MB were analyzed from these HDF5 files. Euchromatin segments were defined as those 1 MB segments that shows more than 10 peaks of H3K27ac chromatin marks, while heterochromatin marks showed enrichment of more than 10 peaks of H3K9me3. Propeller Twist predictions at resolution of 1MB in mouse genome (mm9) was analyzed from BigWig files downloaded from GBShape (Chiu et al., 2015) using Deeptools (Ramirez et al., 2014). For visualization of 3D genome and chromosome structures, PDB files were downloaded from supplementary information provided in study from Stevens et al.(Stevens et al., 2017). The PDB files visualization and programming was done using Pymol. Overlay of chromatin states, surface depth and propeller Twist quartiles was done by

processing PDB files in R and visualization in Pymol. Scripts used for processing are available upon request.

Analysis of CAGE and STARR-seq enhancers

capSTARR-seq data was downloaded from the supplementary data published in the study from Vanhille et al(Vanhille et al., 2015). CAGE Enhancer analysis was downloaded from FANTOM5 Atlas (Andersson et al., 2014). Propeller Twist profiles were overlaid using mean across BED file, centered at mid, using DNAshapeR (Chiu et al., 2016).

Histone modification ChIPseq analysis

Following ChIP-seq were processed in this manner: mES H3K27ac, mES H3K9me3, K562 H3K27ac. The ChIP-sequencing output in FASTQ format was subjected to a quality check using FASTQC v2.6.14 (Andrews). Bowtie v0.12.9 (Langmead, 2010) was used to align the reads uniquely, i.e., each read was maximally aligned to one position, to mm9 genome with UCSC annotations (Rosenbloom et al., 2015). The alignment output files from two biological replicates were merged together after checking for correlations across the replicates using the SAMTOOLS v0.1.19 (Li et al., 2009) merge function. SAMTOOLS v0.1.19 (Li et al., 2009) was used for the alignment file format conversions and sorting of alignment output files. The WIGGLE files for the alignment files were generated using QuasR package (Gaidatzis et al., 2015). The peaks were computed without providing input with MACS v2.0.10.20120913 (Zhang et al., 2008) using the default parameters. The enrichment was calculated by QuasR (Gaidatzis et al., 2015) using the following formulae:

$$Enrichment = log2((\frac{ns}{Ns} * \min(ns, nb) + p) / (\frac{nb}{Nb} * \min(ns, nb) + \mathrm{p}))$$

where ns is the total number of reads that align at the genome level in the ChIP-seq Sample, Ns is the number of reads that aligned the entire ChIP-seq sample, nb is the number of reads that aligned at the genomic level in the input, and Nb is the total number of reads that aligned in the input. P is the pseudocount, which is used to correct the enrichment values at the genomic features with low read counts and was set to 8. For ChIPseq of H3K27ac during time-points of reprogramming, data was downloaded from supplementary information provided in the study by Chen et al.(Chen et al., 2016) All other ChIPseq data was downloaded pre-analyzed from ENCODE (de Souza, 2012).

Transcription factor ChIPseq analysis

Transcription factor ChIPseq data for K562 cells was obtained from ENCODE (de Souza, 2012). ChIPseq data was analyzed in the same way as Histone Modification data given below with specifically restricting to narrow peak calling algorithm from MACS (Zhang et al.,

2008) Transcription factor motifs were obtained from TRANSFAC (Wingender et al., 2000) database. Only those Transcription factors were further analyzed for which motif information were available. Motif enrichment analysis was performed using HOMER (Heinz et al., 2010). Binding sites with ChIPseq peaks centered accross motifs were retained for further DNAshape analysis using DNAshapeR (Chiu et al., 2016). The transcription factors were classified according to families from AnimalTF Database (Zhang et al., 2015). For ChIPseq of Oct4 during time-points of reprogramming, data was downloaded from supplementary information provided in the study by Chen et al. (Chen et al., 2016).

Histone modification ChIP-seq in K562 analysis

Histone modification ChIP-seq data in K562 was obtained from ENCODE (de Souza, 2012) as BAM and peak files. Heatmap and density plot enrichment of Histone modifications was generated using ngs.plot.r (Loh and Shen, 2016; Shen et al., 2014). For the PCA plot of Transcription factor distribution on PCA dimensions as a function of enrichments across various chromatin states was undertaken with data of overlap of Transcription factor peaks with histone modification peaks.

Support Vector Machine and linear and logistic regression analysis

Support vector machine was implemented using e1071 R package 80% of data was used for training of SVM model, while rest 20% of rest was used for testing unless specified. Multiple models as independently mentioned were trained for the reducing chances of overfitting. ROC curves were plotted using ROCR package (Sing et al., 2005). Regression analysis was done using R with similar strategy.

Generating of features for Support vector machine analysis

For classification of enhancer positive and random genomic loci, the corresponding sequences of 2000BP centered on the peak-mid were extracted using BEDTOOLS (Quinlan, 2014)and DNAshape analysis was undertaken using DNAshapeR (Chiu et al., 2016). Propeller Twist values for each base pair step was used as feature set for SVM analysis. For classification of cell-type-cluster specific classification, the feature set included PHASTCons (Felsenstein and Churchill, 1996) derived conservation scores of enhancer sequences from each clusters, TRANSFAC (Wingender et al., 1996) transcription factor motifs enrichments for each enhancer sets, PhastCons derived Conservation scores of the transcription factor motif gene were used.

Conservation and Mutation analysis

For mutation analysis, COSMIC database (Forbes et al., 2017) was used to download

phenotypic and non-phenotypic mutations. The genomic classes in increasing order of mutation density was used by scanning number of mutations occurring in each non-overlapping 2000 BP regions, and stratification by the quartile analysis of the counts. Conservation scores were obtained from PhastCons (Felsenstein and Churchill, 1996)

Programming and scripting

Scripts for data analysis is written in R and PERL and is available upon request.

SUPPLEMENTAL REFERENCES

Andersson, R., Gebhard, C., Miguel-Escalada, I., Hoof, I., Bornholdt, J., Boyd, M., Chen, Y., Zhao, X., Schmidl, C., Suzuki, T., *et al.* (2014). An atlas of active enhancers across human cell types and tissues. Nature *507*, 455-461.

Chen, J., Chen, X., Li, M., Liu, X., Gao, Y., Kou, X., Zhao, Y., Zheng, W., Zhang, X., Huo, Y., *et al.* (2016). Hierarchical Oct4 Binding in Concert with Primed Epigenetic Rearrangements during Somatic Cell Reprogramming. Cell reports *14*, 1540-1554.

Chiu, T.P., Comoglio, F., Zhou, T., Yang, L., Paro, R., and Rohs, R. (2016). DNAshapeR: an R/Bioconductor package for DNA shape prediction and feature encoding. Bioinformatics *32*, 1211-1213.

Chiu, T.P., Yang, L., Zhou, T., Main, B.J., Parker, S.C., Nuzhdin, S.V., Tullius, T.D., and Rohs, R. (2015). GBshape: a genome browser database for DNA shape annotations. Nucleic acids research *43*, D103-109.

de Souza, N. (2012). The ENCODE project. Nature methods *9*, 1046.

Felsenstein, J., and Churchill, G.A. (1996). A Hidden Markov Model approach to variation among sites in rate of evolution. Molecular biology and evolution *13*, 93-104.

Forbes, S.A., Beare, D., Boutselakis, H., Bamford, S., Bindal, N., Tate, J., Cole, C.G., Ward, S., Dawson, E., Ponting, L., *et al.* (2017). COSMIC: somatic cancer genetics at high-resolution. Nucleic acids research *45*, D777-D783.

Gaidatzis, D., Lerch, A., Hahne, F., and Stadler, M.B. (2015). QuasR: quantification and annotation of short reads in R. Bioinformatics *31*, 1130-1132.

Greenbaum, J.A., Pang, B., and Tullius, T.D. (2007). Construction of a genome-scale structural map at single-nucleotide resolution. Genome research *17*, 947-953.

Heinz, S., Benner, C., Spann, N., Bertolino, E., Lin, Y.C., Laslo, P., Cheng, J.X., Murre, C., Singh, H., and Glass, C.K. (2010). Simple combinations of lineage-determining transcription factors prime cis-regulatory elements required for macrophage and B cell identities. Molecular cell *38*, 576-589.

Langmead, B. (2010). Aligning short sequencing reads with Bowtie. Current protocols in bioinformatics *Chapter 11*, Unit 11 17.

Li, H., Handsaker, B., Wysoker, A., Fennell, T., Ruan, J., Homer, N., Marth, G., Abecasis, G., Durbin, R., and Genome Project Data Processing, S. (2009). The Sequence Alignment/Map format and SAMtools. Bioinformatics *25*, 2078-2079.

Loh, Y.H., and Shen, L. (2016). Analysis and Visualization of ChIP-Seq and RNA-Seq Sequence Alignments Using ngs.plot. Methods in molecular biology *1415*, 371-383.

Quinlan, A.R. (2014). BEDTools: The Swiss-Army Tool for Genome Feature Analysis. Current protocols in bioinformatics *47*, 11 12 11-34.

Ramirez, F., Dundar, F., Diehl, S., Gruning, B.A., and Manke, T. (2014). deepTools: a flexible platform for exploring deep-sequencing data. Nucleic acids research *42*, W187-191.

Rosenbloom, K.R., Armstrong, J., Barber, G.P., Casper, J., Clawson, H., Diekhans, M., Dreszer, T.R., Fujita, P.A., Guruvadoo, L., Haeussler, M., *et al.* (2015). The UCSC Genome Browser database: 2015 update. Nucleic acids research *43*, D670-681.

Shen, L., Shao, N., Liu, X., and Nestler, E. (2014). ngs.plot: Quick mining and visualization of next-generation sequencing data by integrating genomic databases. BMC genomics *15*, 284.

Sing, T., Sander, O., Beerenwinkel, N., and Lengauer, T. (2005). ROCR: visualizing classifier performance in R. Bioinformatics *21*, 3940-3941.

Stevens, T.J., Lando, D., Basu, S., Atkinson, L.P., Cao, Y., Lee, S.F., Leeb, M., Wohlfahrt, K.J., Boucher, W., O'Shaughnessy-Kirwan, A., *et al.* (2017). 3D structures of individual mammalian genomes studied by single-cell Hi-C. Nature *544*, 59-64.

Vanhille, L., Griffon, A., Maqbool, M.A., Zacarias-Cabeza, J., Dao, L.T., Fernandez, N., Ballester, B., Andrau, J.C., and Spicuglia, S. (2015). High-throughput and quantitative assessment of enhancer activity in mammals by CapStarr-seq. Nature communications *6*, 6905.

Wiggins, P.A., van der Heijden, T., Moreno-Herrero, F., Spakowitz, A., Phillips, R., Widom,

J., Dekker, C., and Nelson, P.C. (2006). High flexibility of DNA on short length scales probed by atomic force microscopy. Nat Nanotechnol *1*, 137-141.
Wingender, E., Chen, X., Hehl, R., Karas, H., Liebich, I., Matys, V., Meinhardt, T., Pruss, M., Reuter, I., and Schacherer, F. (2000). TRANSFAC: an integrated system for gene expression regulation. Nucleic acids research *28*, 316-319.
Wingender, E., Dietze, P., Karas, H., and Knuppel, R. (1996). TRANSFAC: a database on transcription factors and their DNA binding sites. Nucleic acids research *24*, 238-241.
Zhang, H.M., Liu, T., Liu, C.J., Song, S., Zhang, X., Liu, W., Jia, H., Xue, Y., and Guo, A.Y. (2015). AnimalTFDB 2.0: a resource for expression, prediction and functional study of animal transcription factors. Nucleic acids research *43*, D76-81.
Zhang, Y., Liu, T., Meyer, C.A., Eeckhoute, J., Johnson, D.S., Bernstein, B.E., Nusbaum, C., Myers, R.M., Brown, M., Li, W.*, et al.* (2008). Model-based analysis of ChIP-Seq (MACS). Genome biology *9*, R137.
