# Supplementary material for: Deciphering the Gene Regulatory Landscape Encoded in DNA Biophysical Features
Source: iScience. 2019 Oct 31;21:638–49. doi: 10.1016/j.isci.2019.10.055 (PMC6889597; doi:10.1016/j.isci.2019.10.055)
Supplement: Data S1. Details on DNA Sequences Analyzed by AFM, Related to Figures 1 and S1 [file mmc2.zip › Supplementary folder 1/Sequences for AFM analysis.docx]

**ProT pUC57 plasmids**

- Selected 1000 bp inserts with different ProT properties
- DNA was ordered at BioCat (inserts cloned into pUC57 at HindIII site)
- >5 µg of each construct was delivered with QC info (sequencing of the insert, PvuII RE)
- The following oligos were ordered for PCR amplification (1167 bp, one side biotinylated products).
  - M13_pUCF-bio [Btn]-cccagtcacgacgttgtaaaacg ***dissolve in 267 µL to get 100 µM***
  - M13_pUCR agcggataacaatttcacacagg ***dissolve in 480 µL to get 100 µM***
- Sequence files of the plasmids and PCR products were generated using SerialCloner (xdna files, plasmids also as fasta – in the fasta files the 1167 bp fragment is indicated in CAPITAL)

Note:

(1) Sequence files (plasmids as xdna and fasta, PCR product as xdna) are provided separately in this folder.

(2) The biotin label will be on the 5’ end according to the sequence files.
